# Legitimate and Reliable Determination of the Age-Related Intestinal Microbiome in Young Piglets; Rectal Swabs and Fecal Samples Provide Comparable Insights

**DOI:** 10.3389/fmicb.2019.01886

**Published:** 2019-08-14

**Authors:** R. Choudhury, A. Middelkoop, J. E. Bolhuis, M. Kleerebezem

**Affiliations:** ^1^Host-Microbe Interactomics Group, Department of Animal Sciences, Wageningen University and Research, Wageningen, Netherlands; ^2^Adaptation Physiology Group, Department of Animal Sciences, Wageningen University and Research, Wageningen, Netherlands

**Keywords:** rectal swab, feces, gut microbiota, early life, pig

## Abstract

A prerequisite for reliable microbiota analysis is having an effective and consistent sampling method. Fecal sampling, commonly used to study the intestinal microbiome, might not be suitable in all situations, especially considering the potential difficulties in obtaining fresh feces from young animals. Indeed, this study shows that the success rate of collecting fecal samples from young piglets (<2 weeks of age) was very low. Therefore, we evaluated rectal swabs as an alternative sample type (to feces) for studying porcine microbiome development and performed a comparative analysis of microbiome composition obtained from fresh fecal samples and rectal swabs in 15 healthy piglets at seven (6 piglets) and 20 (9 piglets) days of age. Three samples (fresh feces, rectal swab before and after defecation) were collected from individual piglets and microbiome composition was assessed by 16S rRNA gene sequencing. The results demonstrated that rectal swabs and fecal samples provide similar microbiome composition profiles, with samples clustering predominantly by individual animal rather than sample type. Furthermore, regardless of the sample type, the biological interpretation with respect to microbiota colonization patterns associated with different ages (7 and 20 days) was found to be comparable. Independent of sample type, we observed age-related changes like increasing microbiota diversity and alterations in relative abundances of the phyla *Firmicutes, Bacteroidetes,* and *Fusobacteria*, which was also reflected in consistent family- and genus-level microbiota changes. This study establishes that rectal swabs are a suitable alternative sample type to study the porcine microbiome development in early life, when fecal sampling is challenging.

## Introduction

Early life is considered critical in terms of host (immune and metabolic) development and may offer a unique “window of opportunity” for dietary microbiome modulation (Putignani et al., 2014; Pohl et al., 2015; Schokker et al., 2015). This is supported by the observation that early-life perturbations have been linked to the onset of health consequences like irritable bowel syndrome (IBS), inflammatory bowel disease (Pohl et al., 2015), and neuro-behavioral disorders like attention deficit hyperactivity disorder (ADHD) and depression (O’Mahony et al., 2009; Cenit et al., 2017) in adulthood. It is therefore crucial to accurately determine early-life development of the gut microbiome in relation to intestinal (mucosal) development, to eventually unravel microbiome effects on immune-, metabolic-, and neuro-development. Such knowledge could serve to design and evaluate intervention strategies that are intended to modulate early-life gut microbial colonization, and consequently support healthy development of the host organism.

Animal models are often used to study disease etiology, drug discovery, and to investigate fundamental processes. Porcine models have been proposed as a translational model for the study of developmental consequences of early-life perturbation, and/or nutritional studies in humans (Conrad and Johnson, 2015; Mudd and Dilger, 2017). Such proposition is based on anatomical- (cardiovascular and urinary system, skin, and brain) and functional-similarities (gastrointestinal and immune system) (Guilloteau et al., 2010; Nguyen et al., 2015; Wang and Donovan, 2015; Merrifield et al., 2016) and has been driven by the availability of relevant porcine disease models (diabetes, atherosclerosis, gastric ulcer, and wound healing) (Gieling et al., 2011). However, apart from its value as a translational model for human diseases and fundamental processes, the study of early-life development in pigs is also highly valuable in veterinary and animal sciences that aim to improve animal health and welfare.

Previous studies have demonstrated the relevance of early-life manipulation (*via* nutrition, pre/post-natal antibiotics, and stress) and its effect on porcine microbiome and intestinal development (Zhang, 2014; Schokker et al., 2015, 2018; Leblois et al., 2017; Lewis et al., 2017; Mu et al., 2017; McCormack et al., 2018). In the porcine gut microbiome, *Firmicutes* and *Bacteroidetes* are known to be the predominant phyla, regardless of age (Kim et al., 2011; Chen et al., 2017; Lu et al., 2018). Aging has been associated with the increased abundance of *Firmicutes* and the decreased abundance of *Proteobacteria*, *Fusobacteria,* and *Actinobacteria* (Slifierz et al., 2015; Chen et al., 2017). In commercial pig husbandry, weaning is an abrupt event comprising of a dietary shift from sow milk to usually solid-feed based diets, which poses a challenge to piglets during early-life development. During the preweaning phase, microbiome composition is dominated by milk-associated microbial families like *Bacteroidaceae* and *Lactobacillaceae*, which rapidly changes after weaning when a (largely) plant-based diet is introduced. For instance, *Prevotella*, having a very low abundance in suckling piglets, dramatically increases post-weaning due to availability of plant substrates in the feed (Frese et al., 2015; Mach et al., 2015). The rapidly changing microbiome of young piglet seems to increase in microbial diversity and richness in the suckling phase and gradually stabilizes postweaning (Kim et al., 2011; Frese et al., 2015; Slifierz et al., 2015; Chen et al., 2017).

For comprehensive analyses of microbiota colonization patterns, a longitudinal time-series study with a consistent, easy-to-obtain sampling method is required. Fecal sampling has been commonly used to study the intestinal microbiome of humans and animals. However, a few papers have pointed out the practical challenges and confounders during feces collection and analysis in human microbiome studies. This can range from the potential risk of contamination during sample handling, time-lag in cryopreservation, intermediary sample thawing (e.g., during transport to laboratory), to stool consistency (Budding et al., 2014; Bassis et al., 2017; Vandeputte et al., 2017). Varying storage conditions (4, −20, or −80°C), preservation buffer (e.g., TE Buffer, Stabilization buffer) have been known to induce variation in the outcomes of microbiota composition analyses of the samples. In a few clinical studies, rectal swabs and rectal mucosal biopsies were investigated as an alternative (reproducible) sampling method for studying the intestinal microbiome, but these have mainly focused on the consistency of methods for storing and preserving samples in a human clinical setting (Araújo-Pérez et al., 2012; Budding et al., 2014; Bassis et al., 2017; Jones et al., 2018). For studies focused on non-human subjects, microbiome samples are commonly obtained from intestinal content or scrapings collected from euthanized animals. While this method of sample collection evades microbial composition differences between intestinal content and fecal samples, it is incompatible with repeated sampling schemes from the same individual animal and increases the number of animals needed (Ingala et al., 2018). A few studies have been conducted to decipher early-life porcine gut colonization patterns in which either rectal swabs or feces were used to analyze the microbiome composition (Frese et al., 2015; Mach et al., 2015; Slifierz et al., 2015; Chen et al., 2017; Lu et al., 2018). However, there is limited information available about the consistency of the biologically relevant information obtained from rectal swab or fecal sampling regimes. Burrough et al., 2017 have addressed comparability of porcine microbial profiles from intestinal lumen content and mucosal scrapings from the colon of pigs inoculated with pathogenic bacterial strains that causes swine diarrhea (Burrough et al., 2017). Although this study described differences in microbial profiles deduced from those sample types, the microbiota composition consistently clustered by disease phenotype, illustrating that the overall biological interpretation is conserved in both sample types.

The aim of the present study was to investigate whether rectal swabs are a legitimate alternative sampling method relative to fecal samples in studies addressing age-related alterations (early-life microbiota development) in piglets. In addition, we aimed to evaluate whether the timing of the swab collection relative to the moment of defecation had an influence on the microbiome composition detected. We anticipated that collecting fecal samples in young suckling piglets might pose a practical challenge (Pluske et al., 2007). Therefore, we evaluated the success rate of obtaining fecal samples from piglets during the preweaning period and analyzed whether rectal swabs can serve as a reliable alternative sample type. We found that despite rectal stimulation, it is not practically feasible to consistently obtain fecal samples from young piglets, especially during the first weeks of life. However, the readily-obtained rectal swabs are a suitable alternative sample type, and the moment of swab collection relative to defecation, does not have a major impact on the microbiome composition detected. Thereby, this study validates rectal swabs as a suitable sample type compared to feces, and in addition illustrates that early-life changes in microbiome colonization patterns in piglets can be studied irrespective of the sample type employed.

## Materials and Methods

### Rectal Stimulation and Swab, Fecal Sampling

The Animal Care and Use committee of Wageningen University & Research (Wageningen, The Netherlands) approved the protocol of the experiment. Animals (Top Pi × Topigs 20, both sexes, born from 9 multiparous sows ranging from 1 to 7 parity) were housed in the research facilities of Cargill Animal Nutrition Innovation Center, Velddriel, the Netherlands in conventional (2.2 m × 2.0 m) farrowing pens with sows confined in a farrowing crate. Within 2 days after birth, litter size was standardized to 13–15 piglets by cross-fostering. The piglets were reared on the sow and were provided *ad libitum* access to water and creep feed throughout the suckling period. Details about animal housing and standard procedures have been reported previously (Middelkoop et al., 2018).

To evaluate the success rate of rectal stimulation for obtaining fecal samples in suckling piglets, we randomly selected 84 piglets in total from six different pens and rectally stimulated them at different time-points [day 3 (*n* = 26), day 7 (*n* = 36), day 13 (*n* = 43), or day 20 (*n* = 41)] after birth. Rectal stimulation was performed by inserting the tip of a cotton swab 20–30 mm into the rectum and making small, gentle movements (circular and back- and forward) for up to a maximum of 2 min.

To compare fecal and rectal swab samples, 10 piglets were randomly selected from three pens. Rectal swabs and fecal samples were collected at two time-points at 7 and 20 days old. From each animal, three samples were sequentially collected, a pre-swab (rectal swab before defecation), feces (fecal material collected after rectal stimulation), and a post-swab (rectal swab after defecation). Rectal swabs were obtained by inserting a sterile cotton swab (Puritan Medical, Guilford, ME USA; Cat Number-25-3306-U) 20–30 mm into the rectum and rotating the swab against the bowel wall. Swabs were withdrawn and placed in 5 ml Eppendorf tubes prefilled with 500 μl of phosphate buffered saline (pH 7.0). Fecal material was collected in a sterile container (Sarstedt, Nümbrecht, Germany; Cat number-80.734.311), kept on ice during transport to the laboratory, where they were stored at −20°C until further processing.

### DNA Extraction

DNA was extracted from rectal swabs and feces using the repeated bead beating method (Yu and Morrison, 2004) and the QIAamp DNA Stool Mini Kit (Qiagen, Hilden, Germany), according to manufacturer’s instructions. This method combines lysis by bead beading, DNA precipitation, RNA and protein removal, and DNA purification by QIAamp DNA Stool Mini Kit to obtain the maximum yield from fecal material samples. About 0.1 g of fecal samples and 500 μl of rectal swab solution was used as starting material for DNA extraction. The cells were lysed in Lysing Matrix B tubes prefilled with 0.1 mm silica beads (MP Biomedicals, Santa Ana, California, USA) using FastPrep-24™ (MP Biomedicals, Santa Ana, California, USA) at 5.5 m/s for 3 min (with intermittent cooling on ice in between after every minute). Following the protocol of Yu and Morrison (Yu and Morrison, 2004), RNA and protein were removed from the samples by DNase-free RNase (10 mg/ml, Qiagen, Hilden, Germany) and Proteinase K (Qiagen, Hilden, Germany) treatment. DNA was subsequently purified using the QIAamp DNA Stool Mini Kit (Qiagen, Hilden, Germany) as previously described (Yu and Morrison, 2004). DNA integrity and quantity were determined using a Nanodrop DeNovix DS-11 Spectrophotometer (DeNovix Inc., Wilmington, DE USA) according to manufacturer’s instructions.

### Amplification and Sequencing of 16S rRNA Gene

The V3-V4 region of the bacterial 16S rRNA gene was PCR-amplified using V3F primer (5′-CCTACGGGNGGCWGCAG-3′) and V4R primer (5′-GACTACHVGGGTATCTAATCC-3′), 5′-extended with adapter sequences 5′-TCGTCGGCAGCGTCAGATGTGTATAAGAGACAG-3′ and 5′-GTCTCGTGGGCTCGGAGATGTGTATAAGAGACAG-3′, respectively, which are required for sequencing purposes (see below). PCR reactions were carried out in a Bio-Rad C1000 thermal cycler (Bio-Rad Laboratories, Veenendaal, The Netherlands) using a 50 μl total volume, consisting of 5 μl 10× KOD buffer (Toyobo, Japan), 3 μl MgSO4 (25 mM) 5 μl dNTPs (2 mM each), 1.5 μl V3F primer [10 μM (Eurogentec, Luik, Belgium)], 1.5 μl V4R primer [10 μM, (Eurogentec, Luik, Belgium)], 1.0 μl (0.02 U/μl) KOD hot start DNA polymerase (Toyobo, Japan) and 10 ng (minimum) of template DNA. PCR cycles were programmed with a single initiation cycle of 95°C for 2 min, followed by 25 amplification cycles encompassing denaturation at 95°C for 20 s, annealing at 55°C for 10 s, and elongation at 70°C for 15 s, and was completed by a single elongation step at 72°C for 5 min. Amplicon concentrations were measured by DeNovix DS-11 Spectrophotometer (DeNovix Inc., Wilmington, DE USA) and amplicon size (~550 bp) was verified by agarose (1%) gel electrophoresis. Subsequently, the amplicons were purified from the PCR reaction mixture using MSB Spin PCRapace (STRATEC Molecular, Germany).

Purified amplicons were subjected to extension PCR using barcoded Illumina universal index sequencing adapters prior to sequencing. The samples were sequenced (paired-end) using the Illumina MiSeq system (performed by BaseClear BV, Leiden, The Netherlands), generating FASTAQ sequence files by Illumina Casava pipeline version 1.8.3. Quality assessment was based on Illumina Chastity filtering and FASTQC quality control tool version 0.10.0. A BaseClear in-house filtering protocol was applied for removal of reads containing adapters and/or PhiX control signal.

### Phylogenetic Composition Analysis

The 16S rRNA gene sequencing data was processed and analyzed using CLC Genomics Workbench version 10.1.1 and CLC Microbial Genomics Module version 2.5 (CLC bio, Arhus, Denmark). The paired end reads were merged into one high quality representative by default settings of CLC Workbench (Mismatch cost = 1, Minimum score = 40, Gap Cost = 4, Maximum unaligned end mismatches = 5). The CLC pipeline was used for primer and quality trimming (Trim using quality scores = 0.05; Trim ambiguous nucleotides: maximum number of ambiguities = 2; Discard reads below length = 5). Greengenes 13.5 reference database^1^ (DeSantis et al., 2006) was used for sequence alignment and sequences were binned into operational taxonomic unit (OTUs) based on 97% similarity. The OTU table is further filtered by removing OTUs with low abundance (Minimum combined count = 10), to get a final abundance table for each sample. The phylogenetic tree was constructed using Maximum Likelihood Phylogeny tool based on a Multiple Sequence Alignment of the OTU sequences (100 most abundant OTUs) generated by MUSCLE (Multiple Sequence Comparison by Log- Expectation) tool (Edgar, 2004) in the workbench. The Maximum Likelihood Phylogeny tool determines the probability of the sequences in the tree, using Neighbor Joining as construction method and Jukes Cantor as Nucleotide substitution model. The OTU table was normalized by rarefaction to an even sequencing depth (20,000 reads) in order to remove sample heterogeneity. The rarefied OTU table was used to calculate alpha diversity indices like number of OTUs and Chao1 indices.

### Comparative Microbiota Analysis

To determine diversity shared between two communities (beta diversity), UniFrac metric was used. Weighted UniFrac distance was calculated in CLC workbench and significance was measured by PERMANOVA analysis (permutational multivariate analysis of variance) with 99,999 number of permutations. Log transformation was used as a normalization method for downstream analyses including univariate analysis of taxa relative abundance (Dhariwal et al., 2017)^2^, redundancy analysis and heat maps. Principal component analysis (PCA; unsupervised) and partial redundancy analysis (pRDA; supervised) were performed using CANOCO 5 (Microcomputer Power, Ithaca, NY, USA) according to manufacturer’s instructions (Braak and Smilauer, 2012). The pRDA procedures were performed to analyze the effect of intrinsic experimental variables (age, pen, and gender) separately by removing remaining covariates in the data from the ordination as described in Canoco 5 manual (Braak and Smilauer, 2012). In an RDA plot, arrows pointing in the direction of environmental variable (or confounding factors) correspond to species (OTU/genus/family) that are predicted to be positively correlated with the values for that environmental variable. The key microbial families correlating to 7 and 20 days of age in fecal samples were selected by their response scores in the biplot, obtained by projecting the species points perpendicular to the axes of environmental variables. The same microbial families were visualized in heat maps for rectal swabs to assess sample type differences. To characterize the age-related dynamics of microbial community, heat maps were constructed by hierarchical clustering of microbial families (selected from Redundancy analysis) in Perseus software^3^, where relative abundance values were log_2_ transformed and subsequently normalized by z-score transformation. Euclidean distance was utilized to measure the distance and clustering was conducted using the average linkage method. The pen-related effects on the microbiota were analyzed using only the data obtained at 20 days of age, to obtain a more equal pen-distribution, and to avoid the confounding age-related effect. To perform comparative analysis of OTUs among sample types, OTUs less than 10 reads in pre-swab, feces or post-swab were discarded. Differential taxa abundance (cut off ≤0.01%) between groups were determined by *t*-test and Mann Whitney test *t-*test in GraphPad Software 5.03 (California, USA, www.graphpad.com). Univariate analysis (Kruskal-Wallis test) was used to compare the microbial composition among sample types and Benjamini-Hochberg FDR value (<0.05) was used to correct the multiple testing. Linear discriminant analysis Effect Size (LEfSe) analysis (Segata et al., 2011) was used to identify microbial groups, which were enriched in sample types. The null hypothesis for all statistical tests was rejected at *p* < 0.05.

## Results

### Rectal Stimulation and Fecal Sampling

**Figure 1:**
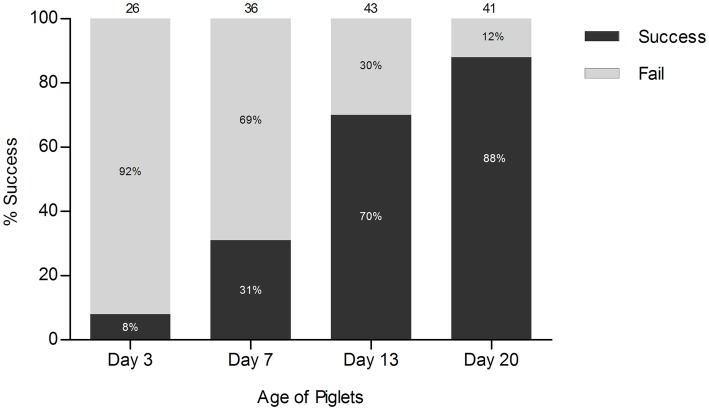
Success rate of rectal stimulation. The numbers at the top of each bar represent the total number of piglets that were stimulated rectally at each time-point.

Feces are the most commonly collected sample type for intestinal microbiota studies. However, sometimes it might be practically challenging to collect such samples, particularly from very young animals. With the aim of understanding, early-life microbiota development in young piglets, we evaluated the success rate of obtaining fecal samples using rectal stimulation on randomly selected piglets from six different pens at four time-points after birth (average 2, 7, 13, and 20 days of age). Rectal stimulation of suckling piglets did not provide fecal material in all cases, and particularly failed in piglets younger than 2 weeks of age, with a success rate of only 8 and 31% at 2 and 7 days of age, respectively (Figure 1). At a later age, rectal stimulation was increasingly successful with success rates increasing to 70 and 88% at 13 and 20 days of age, respectively.

### Fecal and Swab Sample Collection

As mentioned above, rectal stimulation and fecal sample collection success rate in very young (<2 weeks) piglets was low, indicating that an alternative sampling method is required to reliably obtain longitudinal samples for the investigation of early-life porcine microbiome development. Rectal swabs may serve as an alternative sample type for this purpose, and we investigated the comparability of microbiota composition data in rectal swabs and fecal samples. Additionally, we investigated the impact of the timing of rectal swab sampling relative to defecation (before or after), which may also influence the microbiome composition data obtained from the rectal swabs. To this end, we collected a rectal swab before defecation (pre-swab), followed by collection of a fecal sample upon rectal stimulation, and subsequently a rectal swab after defecation (post-swab) from the same piglet to compare microbiota composition results between these sample types. These combined samples were collected from 10 piglets of three pens at 7 (sample 1–10) and 20 (sample 11–20) days of age (Supplementary Table S1). Only complete sample sets (pre-swab, feces, and post-swab) that consistently yielded sufficient DNA to amplify the V3-V4 variable region of the 16S rRNA encoding gene, were analyzed by sequencing (45 samples in total from 15 piglets). This restriction led to the exclusion of four and one sample set (s) collected at 7 and 20 days of age, respectively.

### Microbiota Composition Analysis

Illumina sequencing of the 16S rRNA amplicons generated more than 1.8 million high-quality reads that were classified into OTUs with an average of 41,020 ± 5620 (SD) reads per sample (Supplementary Table S1). The average number of OTUs per sample (after filtering) was 516 ± 22.

**Figure 2:**
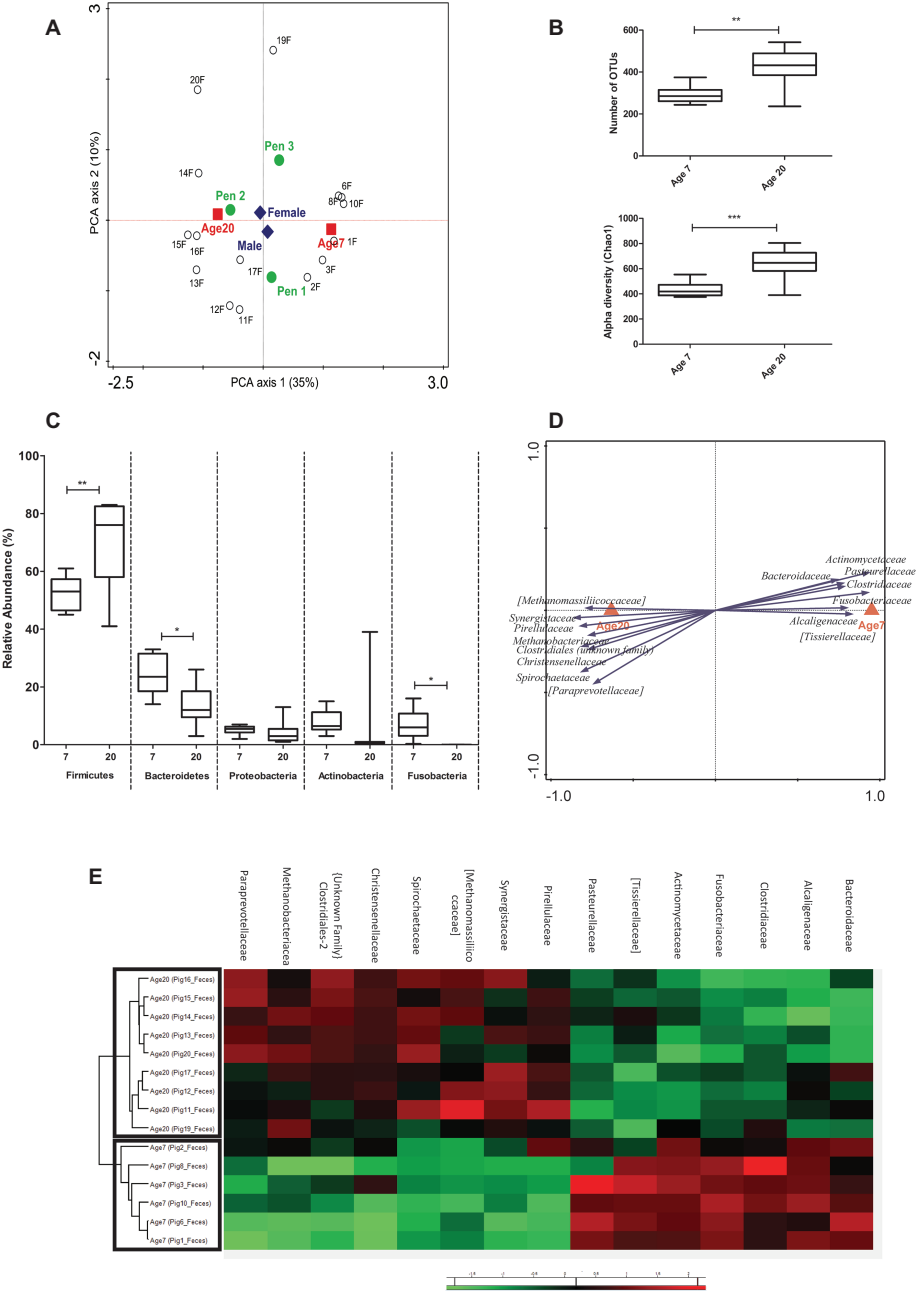
Microbial composition and explanatory variables of fecal samples. **(A)** Principal Component Analysis (PCA) plot showing variation in terms of explanatory variables in the data. Testing single variables by Weighted UniFrac PERMANOVA supports clear separation by age (*p* < 0.001) and pen (*p* = 0.013) but not by gender (*p* = 0.948). **(B)** Alpha diversity (Number of OTUs and Chao1 bias corrected) showing increased diversity at 20 compared to 7 days of age. **(C)** Relative abundance of five phyla (with highest relative abundance) at two ages in fecal samples (Mann Whitney test). **(D)** Partial redundancy analysis (pRDA) for the explanatory variable age (corrected for pen) at family level (RD1 = 40.23% and RD2 = 11.36%). **(E)** Heat map showing relative abundance of 15 most discriminative bacterial families for age. ^*^p < 0.05, ^**^p < 0.01, ^***^p < 0.001.

Since fecal samples are the most commonly used sample type for microbiota composition analysis, we first analyzed the data derived from these samples, focusing first on potential explanatory variables. This analysis revealed a highly significant age effect on the microbiota, an expected pen effect, and a small-sized influence of gender (Figure 2A). Since gender was found to have a limited and non-significant effect in our experiment (seen in RDA analyses; Supplementary Figure S2A), it was excluded as a covariate in further analysis. In contrast, the effect of pen in the microbial composition was significant (Supplementary Figure S2B), which has also been observed in other studies (Mach et al., 2015; Yang et al., 2017). The impact of age on the microbiota was reflected in a significant increase of the microbiota diversity at 20 relative to 7 days of age (Figure 2B). As expected, bacteria classified among the *Firmicutes* and *Bacteroidetes* phyla were the most abundant in all fecal samples, followed by members of the *Proteobacteria* and *Actinobacteria* (Supplementary Figure S1). Comparison of the fecal samples revealed that the relative abundance of the *Firmicutes* increased (52.5 to 69.7%, *p* = 0.010) and *Bacteroidetes* decreased (24.2 to 13.2%, *p* = 0.014) when the piglets aged from 7 to 20 days after birth (Figure 2C). Notably, bacteria classified among the *Fusobacteria* were relatively abundant at 7 days of age, but were not detected (absent) at 20 days of age (*p* = 0.025), whereas *Actinobacteria* displayed high relative abundance variation due to a single animal having a high relative abundance of this phylum at 20 days of age (Figure 2C).

Pen and age-related changes in the microbiota were further investigated by redundancy (RDA) and partial redundancy (pRDA) analyses. The RDA analyses revealed a significant pen effect on the microbiome composition at genus level (Supplementary Figure S2B). The pRDA for age (corrected for pen) revealed a significant association of specific bacterial families with 7 or 20 days of age. The microbiota associated with 7 days of age displayed a higher relative higher abundance of the *Actinomycetaceae*, *Fusobacteriaceae*, *Tissierellaceae, Alcaligenaceae, Clostridiaceae, Pasteurellaceae,* and *Bacteroidaceae*, whereas the microbiota at 20 days of age appeared enriched for *Synergistaceae, Methanobacteriaceae, Spirochaetaceae, Methanomassiliicoccaceae, Christensenellaceae, Pirellulaceae,* and *Paraprevotellaceae* (Figure 2D). Hierarchical cluster analysis performed on the bacterial families associated with age-related microbiota changes, clearly separated 7 and 20 days of age-associated microbiota (Figure 2E), although not all families appeared to be equally discriminating.

These analyses demonstrate that substantial microbiota changes can be detected in fecal samples from piglets during early-life preweaning stages (7 and 20 days of age), and that these changes can be associated with changes in specific bacterial phyla and families. In particular, the family-based analysis led to the detection of a microbial family-signature that clearly discriminates the two ages analyzed here.

### Sample Type Analysis

**Figure 3:**
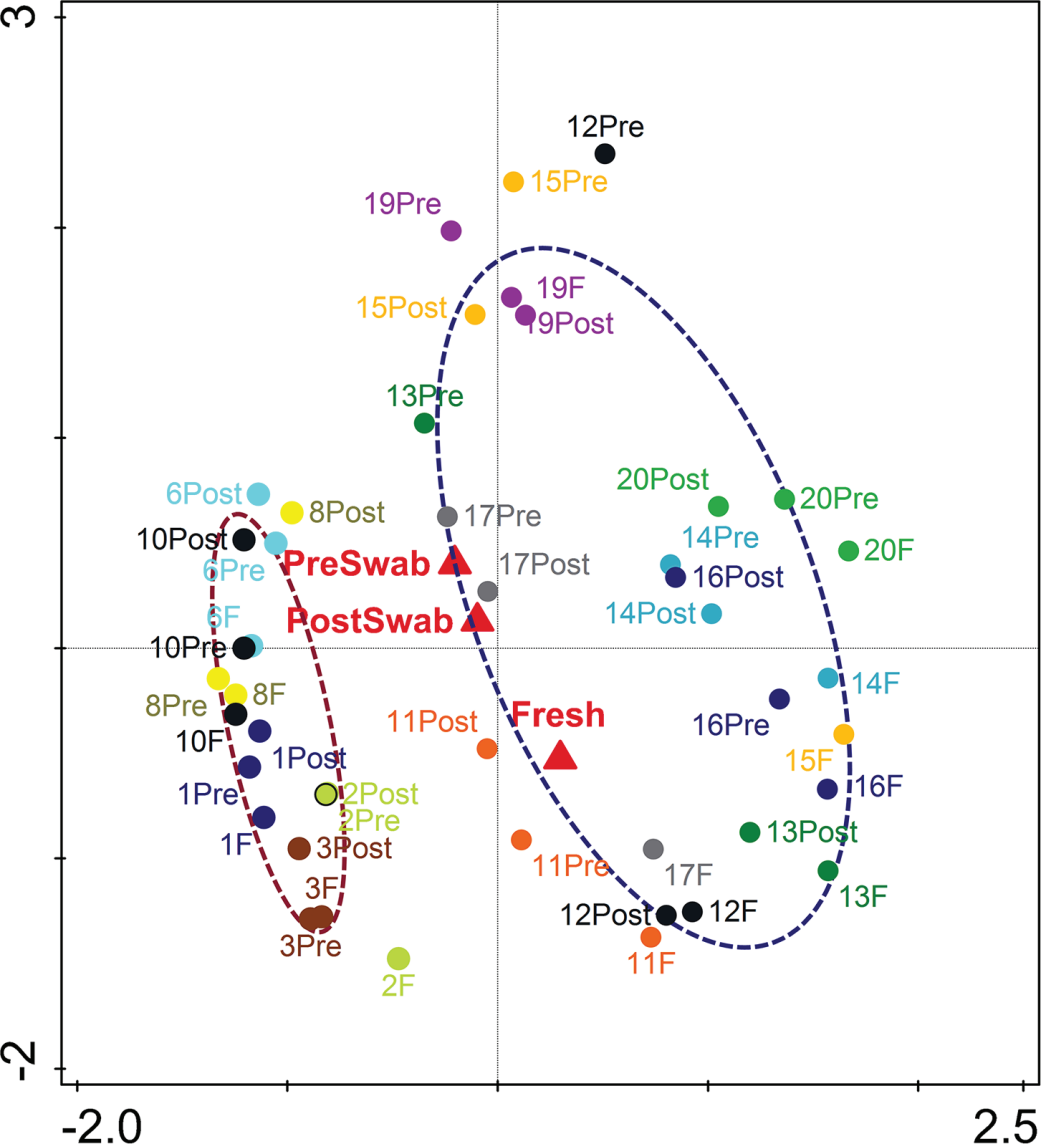
Principal component analysis (PCA) based on OTU level microbiota composition of all samples (PC1 = 26.02%, PC2 = 10.49%). Pre-swab (Pre), Feces (F), Post-swab (Post) are indicated by red triangles; dotted clouds indicate 7 (brown) and 20 days (blue) of age separating age-related microbiota composition irrespective of sample type. The colors denote individual animals.

**Figure 4:**
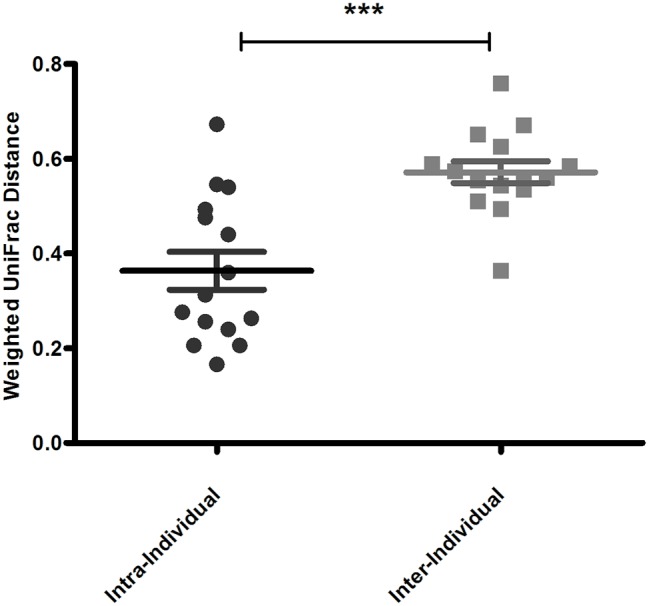
Mean pairwise Weighted UniFrac distance reflecting sample to sample variation within animal (Intra-individual) and between animal (inter-individual). Averaged weighted UniFrac distances indicated that inter-individual variation is greater than intra-individual variation (^***^p < 0.0001).

**Figure 5:**
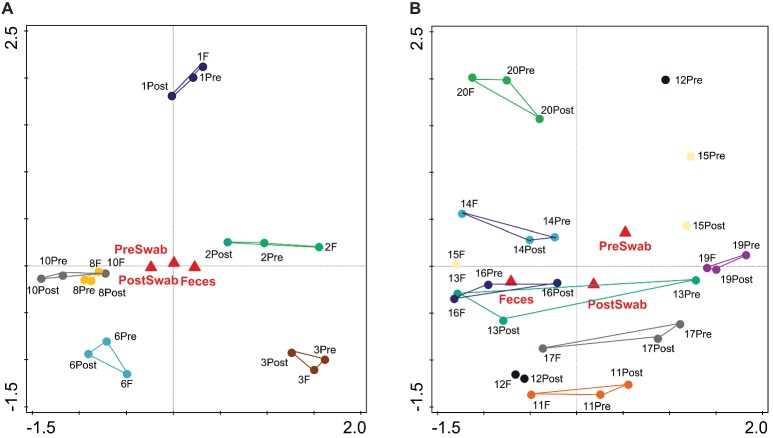
Principal component analysis (PCA) based on OTU level microbiota composition of sample types. Pre-swab (Pre), Feces (F), and Post-swab (Post) are indicated by red triangles **(A)** at day 7 (PC1=26.17%, PC2=18.74%) **(B)** at day 20. (PC1=21.20%, PC2=14.41%). The colors denote individual animals.

An important aim of this study was to investigate whether rectal swabs recapitulate the microbiota composition differences detected in fecal samples. Unsupervised clustering of all samples corroborated that clustering by age is predominant in the data irrespective of the sample type (Figure 3). Comparative analysis of the OTUs identified in each sample type revealed an overlap of 51.42% shared OTUs among the sample types. Importantly, these shared OTUs captured the vast majority of the total microbial population (93–98%) in different sample types (Supplementary Figure S3). Global analysis of alpha and beta diversity failed to detect significant differences between sample types (Supplementary Figure S4). Moreover, weighted UniFrac distance of different sample types (fecal versus pre- and post-swab) obtained from individual animals revealed a substantially higher similarity “between sample types” (intra-individual) as compared to “between animals” (inter-individual) (Figure 4, *p* < 0.0001). In other words, microbiota differences of individual animals are substantially larger than differences between sample types. This inference is also supported by principal component analysis at both ages, showing that samples cluster predominantly by individual animal and not by sample type, although the clustering of three animals/samples was less clear (Figures 5A,B). These disparities in the PCA analysis for these three animals (number 12, 13, and 15) could be explained by a higher relative abundance (although not significant) of a few microbial groups (Figure 5B, Supplementary Figure S5).

**Figure 6:**
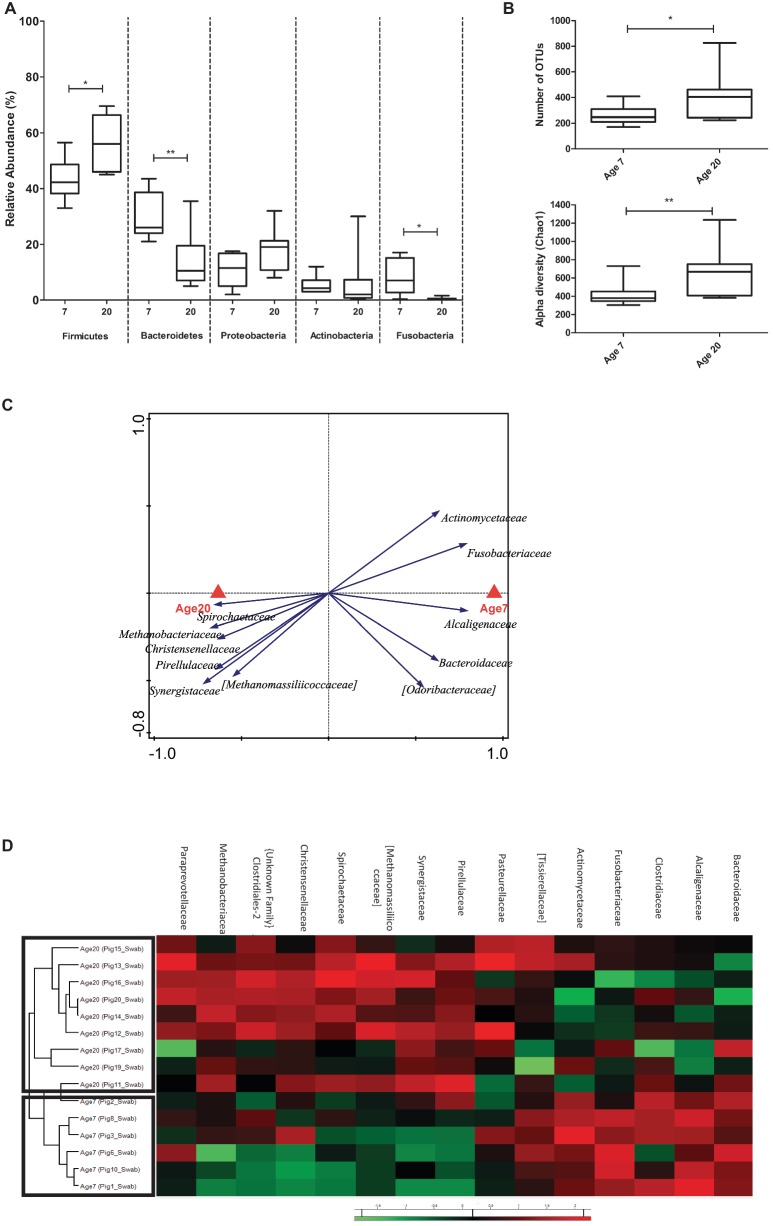
Microbial community composition of rectal swab samples. **(A)** Relative abundance of five phyla (with highest relative abundance) at two ages in rectal swabs (Mann Whitney U-test, ^*^p < 0.05, ^**^p < 0.01). **(B)** Alpha diversity (Chao1 bias corrected) display changing diversity between the two ages. **(C)** Redundancy analysis (partial RDA) of explanatory variable age (corrected for pen) at family level of both the swabs (RD1 = 20.05% and RD2 = 16.79%). **(D)** Heat map showing relative abundance of most discriminative bacterial families for age (same as fecal samples).

Analysis of the rectal swab derived microbiota data (including both pre- and post-swab) established that similar age-related microbiota development changes (phylum-level relative abundance changes) could be detected in the swab samples as compared to the fecal samples (Figure 6A). Analogous to the fecal microbiota changes over time, the swab samples identified increasing *Firmicutes* and decreasing *Bacteroidetes* abundance at day 20 relative to day 7. Similarly, *Fusobacteria* were detected at much higher abundance at day 7 relative to day 20, although they did not disappear completely at this later time point according to swab-derived microbiota. Finally, the abundance of *Actinobacteria* also displayed a high variability between animals at 20 days of age. A notable difference between the swab and fecal sample derived microbiota data was a higher abundance of *Proteobacteria* (averaging above 10% relative abundance) in swab samples as compared to feces (averaging below 5% relative abundance) (Figure 6A).

To further investigate whether swab-sample derived analyses lead to similar biological conclusions as fecal samples, we repeated the analyses that were performed for fecal samples using the swab-derived microbiota data, focusing on age-related changes. Importantly, the swab samples also displayed higher alpha diversity at day 20 compared to day 7 (Figure 6B), and the pRDA for age (corrected for pen) identified most (but not all) of the bacterial families and genera that were associated with day 7 or day 20 in fecal samples (Figure 6C, Supplementary Figure S6). Furthermore, hierarchical cluster analysis performed on these bacterial families separated day 7 and day 20 microbiota samples similar to what was observed with the fecal samples (Figure 6D). However, the bacterial families of *Pasteurellaceae*, *Tissierellaceae*, *Clostridiaceae*, and *Paraprevotellaceae* were not identified as discriminatory variables (apparently less discriminating) in swab analyses, and were included in the hierarchical clustering (Figure 6D) for reasons of comparison with the fecal analyses.

**Figure 7:**
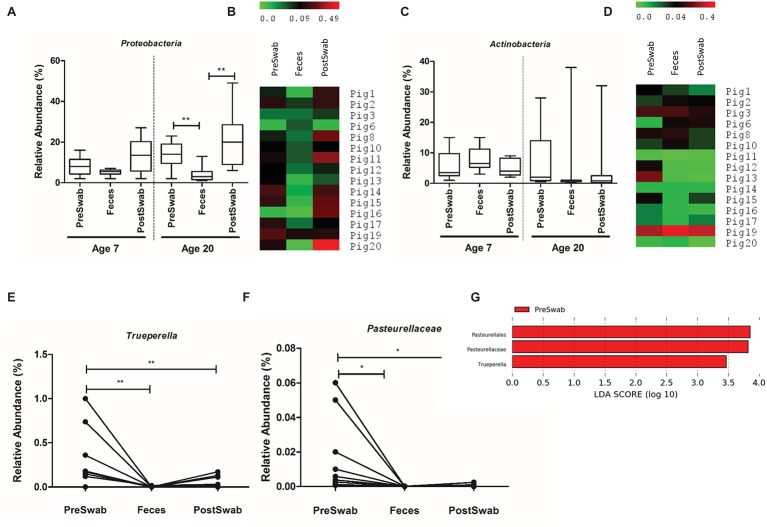
Microbial community composition of sample types. **(A,C)** Relative abundance of *Proteobacteria* and *Actinobacteria* at day 7 and day 20. **(B,D)** Heat map of relative abundance of *Proteobacteria* and *Actinobacteria* at day 20 of all animals. **(E,F)** Relative abundance of the *Trueperella* genus and *Pasteurellaceae* family at day 20 among sample types. **(G)** LEfSe analysis identified *Trueperella* genus and *Pasteurellaceae* family as the only differentially abundant taxa among sample types (^*^p < 0.05, ^**^p < 0.01).

Comparison of different sample types revealed no significant dissimilarities (in relative abundance) at 7 days of age, but detected several differences in the samples obtained at 20 days of age. The relative abundance of the *Proteobacteria* phylum was higher in both pre- and post-swab samples compared to feces, in almost all animals (Figures 7A,B), whereas the post- and pre-swab samples obtained at 20 days of age detected higher and lower relative abundances of *Firmicutes* and *Fusobacteria*, respectively (Supplementary Figure S7). Importantly, the high-level of variation in *Actinobacteria* relative abundance was detected by each of the sample types and was consistently associated with high *Actinobacteria* in a single animal (Figures 7C,D). These analyses illustrate a high level of congruency of the biological conclusions drawn from microbiota analyses using these different sample types.

To refine this conclusion, we performed univariate tests at phylum, family and genus-level relative abundances to compare the three sample types. Only *Trueperella* (genus level) and *Pasteurellaceae* (family level) were significantly more abundant in pre-swab compared to feces and post-swab (Figures 7E–G), although relative abundance of *Pasteurellaceae* was very low (<0.01%). Other microbial families like *Staphylococcaceae* and *Aerococcaceae* were found to be higher in pre-swab samples of few animals, although this was not significant (Supplementary Figure S5). These observations suggest that the microbiota composition in post-swab samples is slightly more comparable to fecal samples, than pre-swab samples. Finally, we assessed the rectal pre- and post-swabs separately to investigate whether the timing of the swab relative to defecation had an effect on rectal swab microbiota composition. This analysis revealed that pre- and post-swab samples generate comparable age-related microbiota composition information (Supplementary Figures S8, S9) and cannot be distinguished on basis of community richness, beta diversity, or differential taxa abundance (Supplementary Figure S4, Figure 7).

## Discussion

Increased insight in early-life intestinal microbiota colonization patterns in piglets is crucial in the design of intervention strategies that aim to modulate developmental processes to improve health of the host organism at later stages of life. Reliable and standardized sampling procedures are required to expand our insights in microbiome colonization during the preweaning stage of life of piglets. The purpose of this study was mainly to evaluate sample types, with a secondary objective to verify whether the age-related differences can be identified irrespective of the sampling procedure. To address this, the present study explored three questions: (1) What is the success rate of obtaining fresh feces from young piglets?; (2) Do rectal swabs provide a reliable alternative sample type for the canonically used fecal samples to study age-related microbiota composition and diversity?; (3) Does the timing of obtaining the rectal swab sample, relative to defecation influence the microbiota profile detected in the rectal swabs?

Rectal stimulation was used to assess the success rate of obtaining feces in young piglets, showing that fecal sample collection from piglets younger than 2 weeks of age is unreliable. Therefore, to obtain a comprehensive overview of microbiota colonization from an early age onward would be difficult if one would rely on fecal sampling. Rectal swabs can provide an alternative sampling choice, although we also observed a higher frequency of technical problems like low DNA yield in swab samples. This is possibly due to the relatively low amount of intestinal material obtained by rectal swab sample at a younger age. However, this hurdle can be overcome by reducing the volume of buffer (PBS) in which the swabs are collected (data not shown) to maintain the maximum possible microbiota biomass concentration and thus increase success rate in downstream microbiota analysis.

The primary aim of this article was to evaluate the similarities and dissimilarities among the sample types (pre-swab, feces and post-swab) obtained from individual animals at two time points (7 and 20 days of age). Although several differences were observed between feces and rectal swabs, these differences were limited to only few microbial groups/taxa. Our comparisons did not reveal differences in microbial richness between the swab and fecal samples. Importantly, our analyses clearly establish that the individualized microbial signature detected in each animal was reproducibly observed and independent of the sample type. Only a few studies have compared microbiome composition of sample types, like rectal swabs, feces and/or mucosal biopsies and/or scrapings (Araújo-Pérez et al., 2012; Budding et al., 2014; Bassis et al., 2017; Jones et al., 2018) in humans. The findings of these studies are largely in agreement with our finding that the microbiome composition can vary (slightly) between sample types (fecal versus swab samples), but the individual animal signature is detected consistently over time. Intriguingly, the swabs we collected prior to defecation (pre-swab) were significantly enriched in taxa belonging to the *Trueperella* and *Pasteurellaceae*, while these bacterial groups were also enriched in the swab samples collected after defecation (post-swab), albeit not significantly. These microbial groups have been associated with mucosal adherence and can thrive in both anaerobe and aerobic conditions (Jacques and Paradis, 1998; Machado and Bicalho, 2014). This observation suggests that swab samples (particularly those that are taken prior to defecation) include a representation of the mucosa-adherent microbiota, with higher abundance of several oxygen-tolerant microbial taxa compared to the microbes residing in the anaerobic intestinal lumen that are detected in fecal material (Jones et al., 2018). This may imply that although fecal samples are considered the ‘gold-standard’ for microbiota analysis, rectal swabs may more adequately represent both luminal and mucosa adherent microbes.

Our analyses illustrated the conservation of the age-dependent microbiota composition changes in fecal and rectal swab samples. The prominent differences in microbiota composition at 7 and 20 days of age, clearly established age-related changes that included increasing community diversity over time and alterations in relative abundance of several phyla e.g. *Firmicutes*, *Bacteroidetes,* and *Fusobacteria*. These changes in microbiome development are consistent with previous studies (Slifierz et al., 2015; Chen et al., 2017; Lu et al., 2018) where these bacterial groups rapidly changed in developing piglets. In our study, a higher relative abundance of *Proteobacteria* was observed in the rectal swab samples compared to the fecal samples. Remarkably, a similar observation has been reported when comparing swab and fecal samples obtained from koala (Alfano et al., 2015). Notably, it has been proposed that the observed abundance of *Proteobacteria* may be influenced by the method used for sample storage (Tris-EDTA buffer or fecal collection tubes with stabilizing medium) (Vandeputte et al., 2017), which suggests that the difference in collection methods for fecal and rectal swab samples could have contributed to the difference in observed *Proteobacteria* relative abundance.

Besides the prominent age-related microbiota effects reported here, pen was an additional environmental variable that was associated with specific microbiota differences. Pen-associated effects could be related to (a combination of) various undistinguishable differences, including a direct environmental effect (same pen), a maternal effect (same uterus, milk, sow), or a host-genetics effect. Our study did not detect a gender-associated microbiota difference, which may not be expected in piglets of this young age where gender-specific development is not yet very pronounced (Reiland, 1978; Medland et al., 2016). Importantly, our study was not designed to address the influence of other environmental variables like pen or gender, and as a consequence the sample distribution over different pens and gender was not very comparable. Study designs to investigate pen and gender-associated microbiota effects require a different design with probably larger numbers of animals to reach significant and meaningful conclusions.

Overall, our study illustrates that despite some differences among the sample types, the biological conclusions drawn are conserved in the three sample types we collected. Furthermore, multivariate analyses (PCA and hierarchical clustering) shows that clustering of samples occurs predominantly per animal rather than sample type, illustrating that sample type is not a major source of variation and is overruled by microbiota signatures of the individual animals. Despite the limited sample size and the unequal distribution of samples over the two time points (6 versus 9) we did succeed to detect consistent and significant age related microbiota signatures that were independent of the sample type used. Thereby this study underpins the reliability of early-life microbiota colonization analyses using rectal swabs. This is particularly relevant because obtaining fresh fecal samples from young animals can be challenging. Future studies with increased numbers of animals and including specific interventions can expand these findings of intestinal colonization in early life and may answer questions related to its susceptibility to modulation, for example by diet, and how such modulation can affect development of the host.

## Data Availability

Raw sequences can be found on SRA-NCBI (Sequence Read Archive-National Center for Biotechnology Information) database under the SRA accession number PRJNA534450.

## Ethics Statement

The Animal Care and Use committee of Wageningen University and Research (Wageningen, The Netherlands) approved the protocol of the experiment.

## Author Contributions

JB and MK conceived the study and acquired funding. RC and AM applied for Ethical Committee approval, collected biological samples, and conducted the experimental investigation. RC processed samples, analyzed sequencing data, statistics, and prepared figures and tables. RC and MK interpreted data and wrote the original draft of the manuscript. All authors discussed the results, commented on the manuscript and approved the final manuscript.

### Conflict of Interest Statement

The authors declare that the research was conducted in the absence of any commercial or financial relationships that could be construed as a potential conflict of interest.

## Footnotes

^1^http://greengenes.lbl.gov/Download/

^2^www.microbiomeanalyst.ca/

^3^http://www.maxquant.org/
