# Supplementary material for: Legitimate and Reliable Determination of the Age-Related Intestinal Microbiome in Young Piglets; Rectal Swabs and Fecal Samples Provide Comparable Insights
Source: Front Microbiol. 2019 Aug 14;10:1886. doi: 10.3389/fmicb.2019.01886 (PMC6702655; doi:10.3389/fmicb.2019.01886)

Supplementary material for:

Legitimate and reliable determination of the age-related intestinal microbiome in young piglets; rectal swabs and fecal samples provide comparable insights

R. Choudhury [1], A. Middelkoop [2], J.E. Bolhuis[2], M. Kleerebezem[1*]

[1]Host-Microbe Interactomics Group, Department of Animal Sciences, Wageningen University & Research, Wageningen, The Netherlands
[2]Adaptation Physiology Group, Department of Animal Sciences, Wageningen University & Research, Wageningen, The Netherlands

Table S1: Table with sample information, read and OTU counts. Number of reads after quality control and removal of chimeric sequences. Number of OTUs at an identity threshold of 97%. Pre=Pre-Swab; Fresh=Feces, Post= Post-Swab .

Animal	Age	Sample	Total Number of reads	Reads in OTU	Number of OTUs
Pig 1	7 days	1_a_Pre	64856	46461	400
Pig 1	7 days	1_b_Fresh	51335	35088	370
Pig 1	7 days	1_c_Post	61620	47897	330
Pig 2	7 days	2_a_Pre	61549	47488	505
Pig 2	7 days	2_b_Fresh	68932	42785	538
Pig 2	7 days	2_c_Post	50107	36861	358
Pig 3	7 days	3_a_Pre	60780	39727	435
Pig 3	7 days	3_b_Fresh	49371	32034	393
Pig 3	7 days	3_c_Post	57895	43054	463
Pig 6	7 days	6_a_Pre	53275	42209	302
Pig 6	7 days	6_b_Fresh	63850	40993	358
Pig 6	7 days	6_c_Post	58549	44955	386
Pig 8	7 days	8_a_Pre	61275	40263	377
Pig 8	7 days	8_b_Fresh	56143	34577	379
Pig 8	7 days	8_c_Post	61297	47089	660
Pig 10	7 days	10_a_Pre	63996	41900	350
Pig 10	7 days	10_b_Fresh	64251	38930	411
Pig 10	7 days	10_c_Post	58679	43788	338
Pig 11 (Pig1_Age20)	20 days	11_a_Pre	64456	48163	471
Pig 11 (Pig1_Age20)	20 days	11_b_Fresh	52314	32978	525
Pig 11 (Pig1_Age20)	20 days	11_c_Post	63502	49349	520
Pig 12 (Pig2_Age20)	20 days	12_a_Pre	54479	31076	847
Pig 12 (Pig2_Age20)	20 days	12_b_Fresh	59355	35360	572
Pig 12 (Pig2_Age20)	20 days	12_c_Post	53176	33136	579
Pig 13 (Pig3_Age20)	20 days	13_a_Pre	67458	45113	395
Pig 13 (Pig3_Age20)	20 days	13_b_Fresh	53850	32569	658
Pig 13 (Pig3_Age20)	20 days	13_c_Post	59725	43014	641
Pig 14	20 days	14_a_Pre	48248	30951	593
Pig 14	20 days	14_b_Fresh	61313	40682	672
Pig 14	20 days	14_c_Post	53789	36363	628
Pig 15	20 days	15_a_Pre	59364	41193	805
Pig 15	20 days	15_b_Fresh	55591	33321	615
Pig 15	20 days	15_c_Post	60832	47610	705
Pig 16 (Pig6_Age20)	20 days	16_a_Pre	63236	39146	651
Pig 16 (Pig6_Age20)	20 days	16_b_Fresh	67263	37811	594
Pig 16 (Pig6_Age20)	20 days	16_c_Post	57183	44139	659
Pig 17	20 days	17_a_Pre	65898	52736	420
Pig 17	20 days	17_b_Fresh	57385	37528	603
Pig 17	20 days	17_c_Post	62514	48299	422
Pig 19	20 days	19_a_Pre	60277	43478	373
Pig 19	20 days	19_b_Fresh	75951	50056	380
Pig 19	20 days	19_c_Post	55105	38751	360
Pig 20 (Pig10_Age20)	20 days	20_a_Pre	69700	46683	785
Pig 20 (Pig10_Age20)	20 days	20_b_Fresh	61750	38903	716
Pig 20 (Pig10_Age20)	20 days	20_c_Post	56498	41380	695

Fig. S1: Taxonomic composition of the 16S rRNA samples. Stacked bar plot depicting relative microbial abundances of all samples at phyla level. Pig 1- 10 are 7 days old (time-point 1) and Pig 11 - 20 are 20 days old (time-point 2); Pre=Pre-Swab; Fresh=Feces, Post= Post-Swab. The figure highlights individual variation captured irrespective of the sample type.

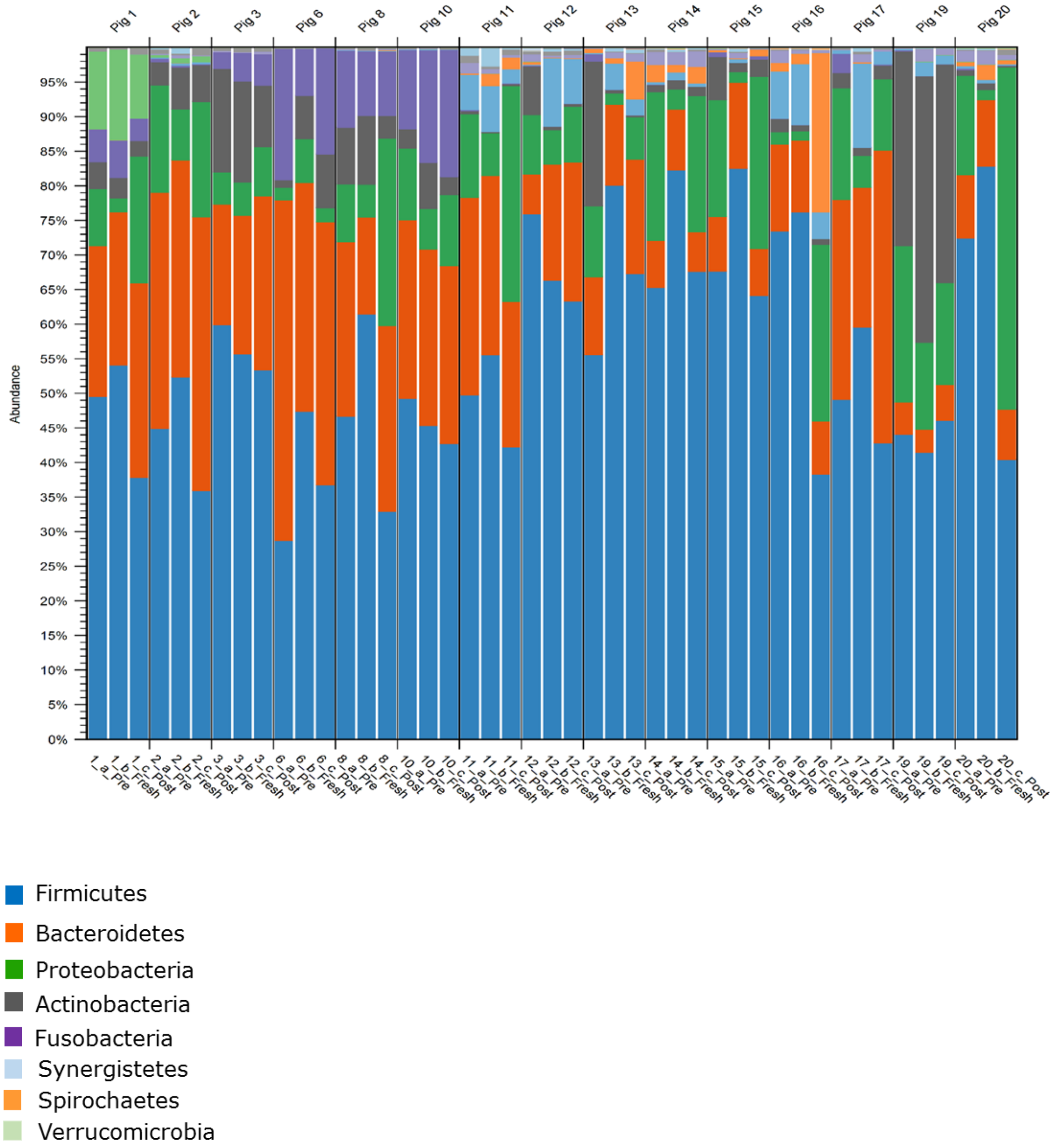

Fig. S2: Biplot of redundancy analysis (RDA) of environmental variables in fecal samples (day20 only) at genus level. **A.** Gender **B.** Pen. Constrained explanatory variables are indicated by triangles. Top right shows the p-value of Monte Carlo Permutation testing. F = fecal Sample, Number = animal ID.

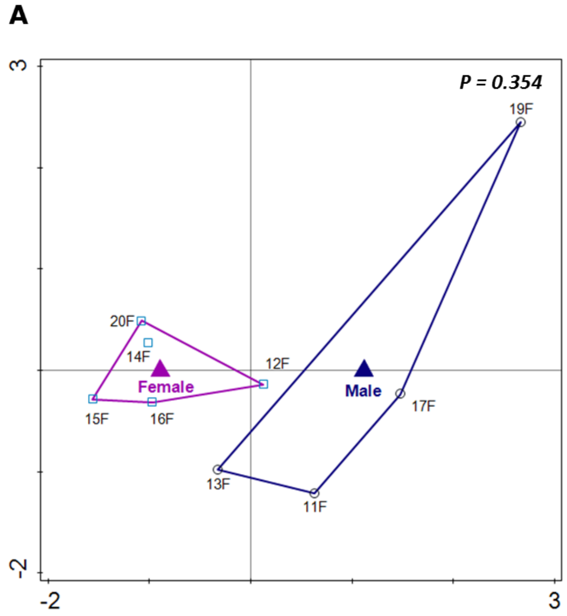

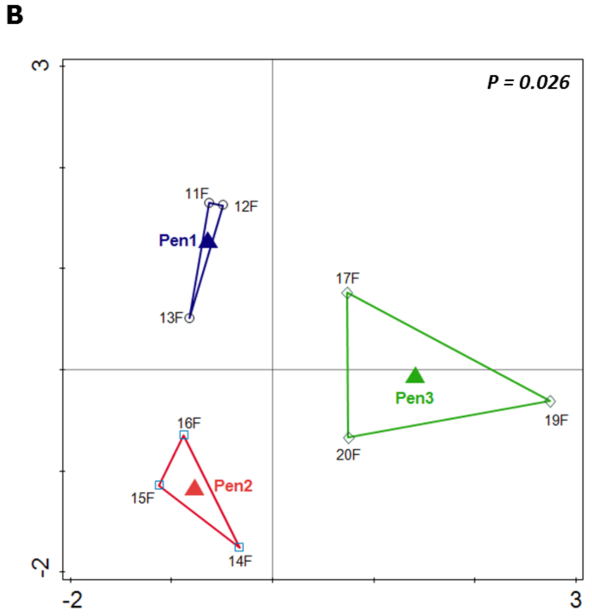

Fig. S3: Venn diagram of bacterial communities with shared and unique operational taxonomic units with at least 10 reads (total OTUs found 1334) in feces, pre-swab or post-swab samples. The percentage within the diagram represents the percentage of the total OTUs unique and shared among the sample types. The percentages (in red) shown outside the diagram represent the relative abundance captured by the 686 shared OTUs.

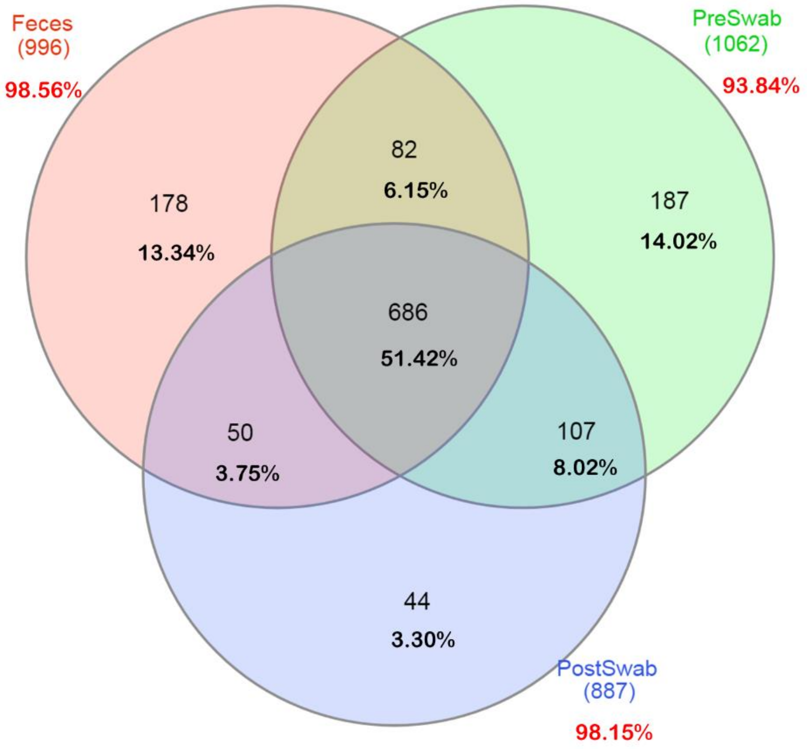

Fig. S4: Alpha and Beta diversity of sample types **A.** Number of species and **B.** Chao1 bias corrected. **C.** PERMANOVA analysis of Weighted UniFrac of sample types at two time-points.

A

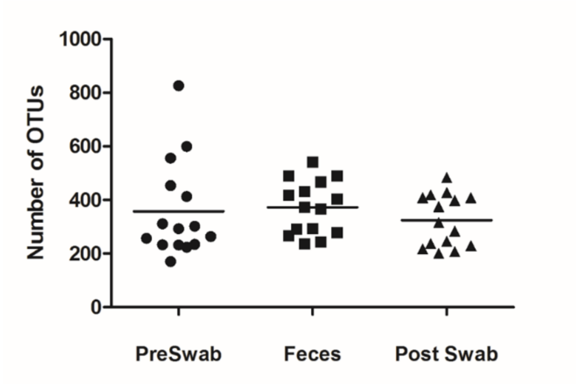

B

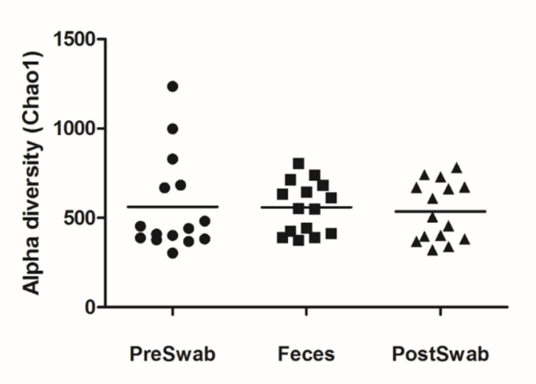

C

PERMANOVA of Weighted UniFrac	Age d7	Age d20
Pre-Swab vs Faeces	P=1.00	P=0.317
Faeces vs Post-Swab	P=1.00	P=1.00
Pre-Swab vs Post-Swab	P=1.00	P=0.32

Fig. S5: Differential abundance of microbial families in three animals (not significant).

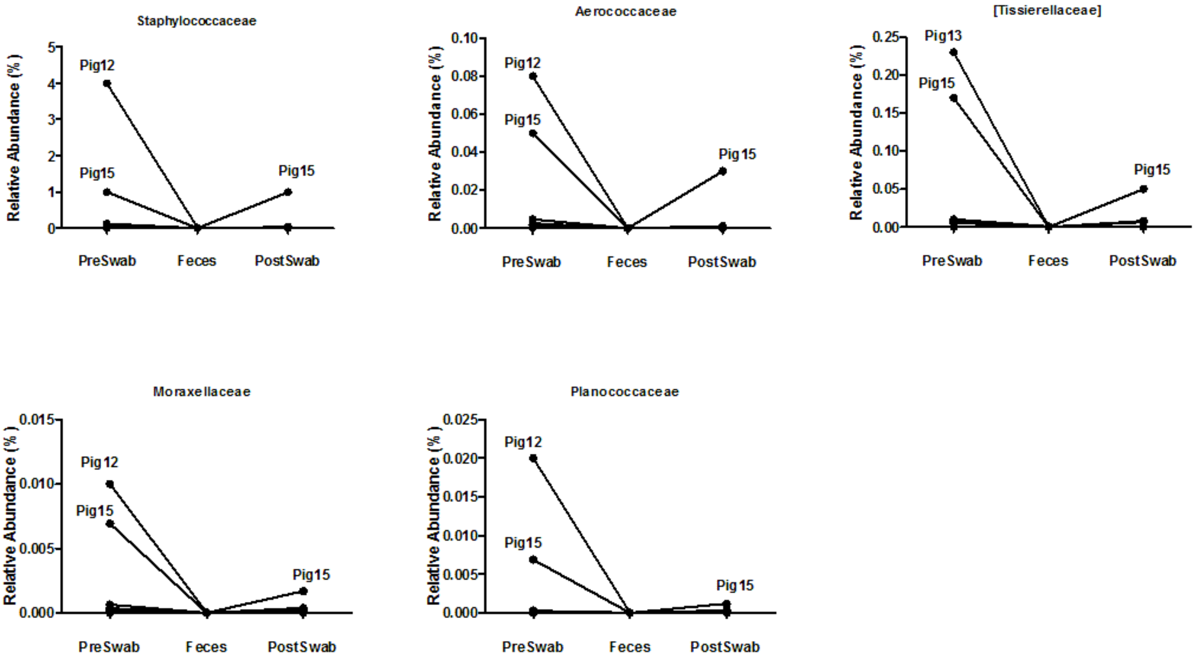

Fig. S6: Partial redundancy analysis (pRDA) for the explanatory variable age (corrected for pen) at genus level. A. Feces (RD1 = 38.85% and RD2 = 10.83%). B. Rectal swabs (RD1 = 16.64% and RD2 = 15.59%)

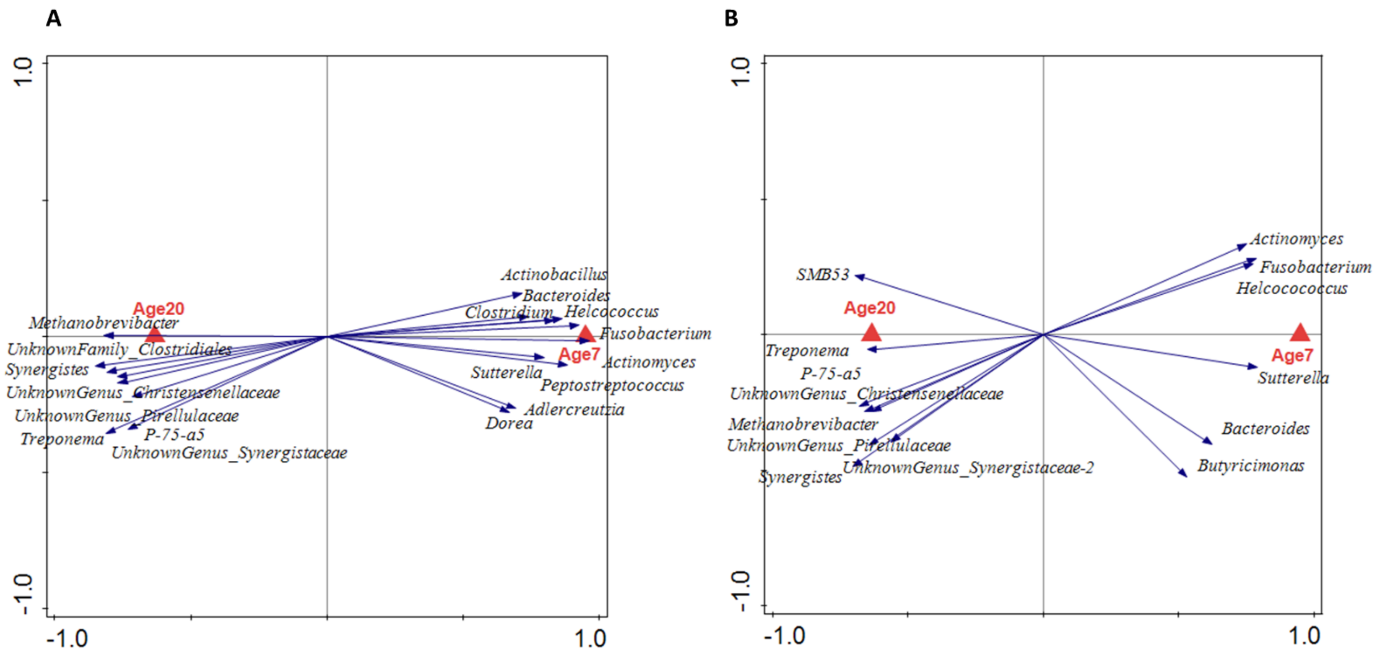

Fig. S7: Relative abundance of sample types (phylum level) at both time-points (the age is indicated in “days after birth”).

*: p<0.05, **: p<0.01.

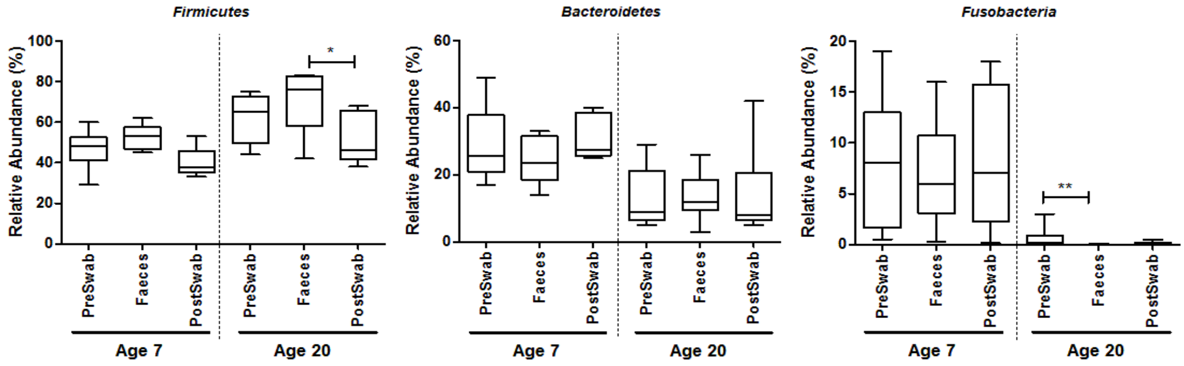

Fig. S8: Principal component analysis of explanatory variables and Redundancy analysis (partial RDA) of age variable (corrected for pen) for **A, C.** Pre-swab samples and **B, D.** Post-swab Samples.

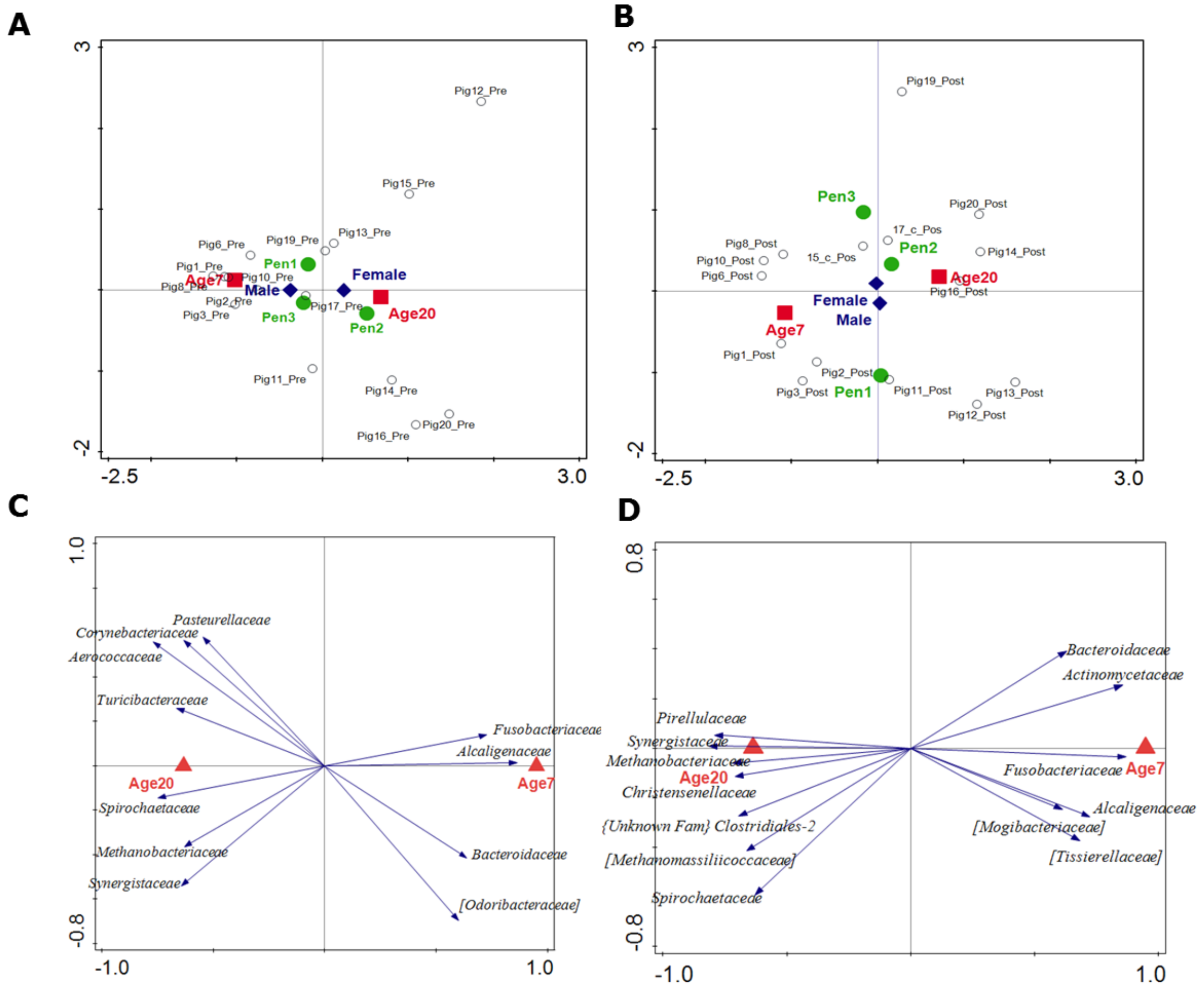

Fig. S9: Heat map showing relative abundance of most discriminative bacterial families for age variable (corrected for pen) for **A.** Pre-swab and **B.** Post-swab samples.

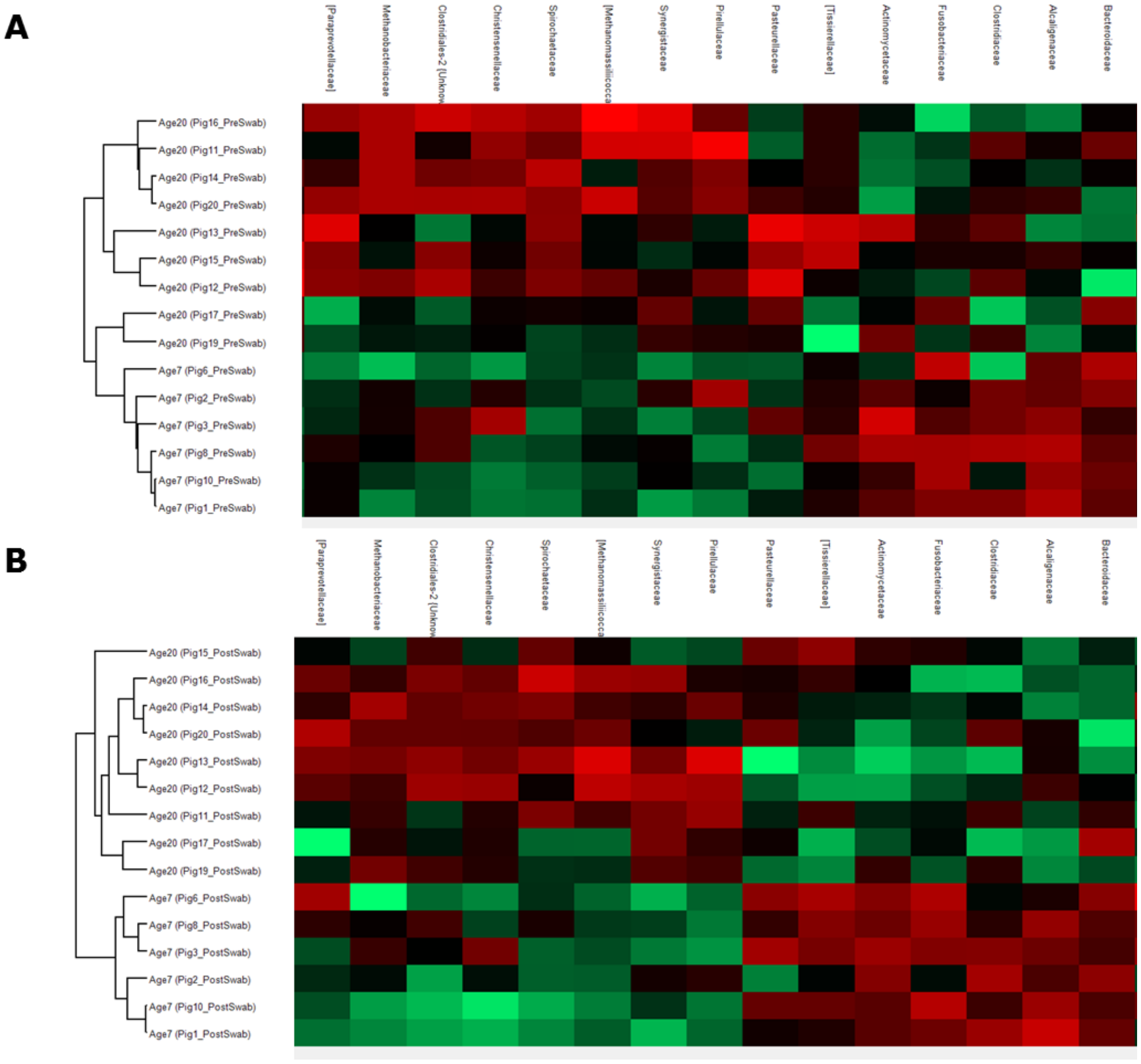

Supplement: Supplementary file 1 [file Presentation_1.pdf]
